# Assessing the Applicability of a Partial Alcohol Reduction Method to the Fine Wine Analytical Composition of Pinot Gris

**DOI:** 10.3390/foods14152738

**Published:** 2025-08-05

**Authors:** Diána Ágnes Nyitrainé Sárdy, Péter Bodor-Pesti, Szabina Steckl

**Affiliations:** 1 Department of Oenology, Institute for Viticulture and Oenology, Hungarian University of Agriculture and Life Sciences, Ménesi Street 45, H-1118 Budapest, Hungary; nyitraine.sardy.diana.agnes@uni-mate.hu (D.Á.N.S.); steckl.szabina@uni-mate.hu (S.S.); 2 Department of Viticulture, Institute for Viticulture and Oenology, Hungarian University of Agriculture and Life Sciences, Villányi Street 29-43, H-1118 Budapest, Hungary

**Keywords:** climate change, alcohol content, fine analytics

## Abstract

Climate change has a significant negative impact on agriculture and food production. This trend requires technological development and the adaptation of new technologies in both the grapevine production and winemaking sectors. High temperatures and heat accumulation during the growing season result in faster ripening and a higher sugar content, leading to a higher alcohol content during fermentation. The negative consequences are an imbalanced wine character and consumer reluctance, as lower alcoholic beverages are now in high demand. Over the last decade, several methods have been developed to handle this impact and reduce the alcohol content of wines. In this study, we used the MASTERMIND^®^ REMOVE membrane-based dealcoholization system to reduce the alcohol concentration in of Pinot gris wines from 12.02% *v*/*v* to 10.69% *v*/*v* and to investigate the effect on analytical parameters in three steps (0.5%, 1%, and 1.5% reductions) along the treatment. To evaluate the impact of the partial alcohol reduction and identify correlations between the wine chemical parameters, data were analyzed with ANOVA, PCA, multivariate linear regression and cluster analysis. The results showed that except for the extract, sugar content and proline content, the treatment had a significant effect on the chemical parameters. Both free and total SO_2_ levels were significantly reduced as well as volatile acid, glycerol and succinic acid levels. It must be highlighted that some parameters were not differing significantly between the untreated and the final wine, while the change was statistically verified in the intermediate steps of the partial alcohol reduction. This was the case for example for n-Propanol, i-Amylalcohol, Acetaldehyde, and Ethyl acetate. The multivariate linear regression model explained 18.84% of the total variance, indicating a modest but meaningful relationship between the alcohol content and the investigated analytical parameters. Our results showed that even if the applied instrument significantly modified some of the wine chemical parameters, those changes would not influence significantly the wine sensory attributes.

## 1. Introduction

### 1.1. Climate Change Induced High Sugar and Alcohol Contents

Climate change has caused a considerable increase in the global surface temperature, resulting 1.09 °C higher values in 2011–2020 than in 1850–1900 [1]. In line with this international trend, local changes in Hungary have shown that the average temperature has increased by 1.15 °C compared to the last century. Over the period 1901–2017, springs and summers warmed the most, at 1.34 °C and 1.25 °C, respectively, while the smallest temperature increase was recorded in autumn, at 0.86 °C [2]. Several strategies have emerged to mitigate the effect according to steps linked to energy, transport, land use, and agriculture [3]. The CAP Strategic Plan Hungary (2023–2027), for example, highlights that all elements of the viti-viniculture sector need to be improved to meet the changing consumer needs and enable the use of the latest technologies. This strategy is in line with the consequences of the climate change, as grapevine (*Vitis vinifera* L.) development, yield, and quality are highly influenced by climatic parameters such as radiation, humidity, rainfall, and temperature. This makes viticulture one of the most vulnerable agricultural sectors to climate change, since the berry composition largely determines the quality and sensory characteristics of the wines produced [4]. Recent studies have shown that one of the main consequences associated with increased ambient temperatures is a shift in timing and shortening of phenological stages [5,6,7]. For example, van Leeuwen and Darriet [8] emphasized that the changing climate shifts ripening earlier while simultaneously altering the berry aroma composition. More particularly, Sandras and Moran [9] found that at elevated temperatures, the berry anthocyanin and sugar levels become decoupled, which could cause an unfavorable balance between the color and alcohol content in the wine. Furthermore, high temperatures during the initial phase of the berry development decrease the production of secondary metabolite components such as phenolic and aromatic compounds [10,11].

The high alcohol content of wines may not only lead to disharmonious products, which may result in reduced consumption, but it may also lead to the loss of further markets as consumer demand shifts toward lower-alcohol beverages. Recently, there has been an increasing trend toward consumers choosing lower-strength drinks (more broadly, 9–13 *v*/*v*% ethanol) and low-alcohol wines (0.5–2 *v*/*v*%) [12]. Increasing health and safety awareness and global initiatives to reduce alcohol consumption are the reasons to produce lower alcohol wines that are attractive to wine drinkers [13]. Meillon et al. [14] and Thompson and Thompson [15] found that people were motivated to consume low-alcohol beverages for calorie and weight control, and that they perceived these low-alcohol beverages as an alternative to standard alcoholic drinks. However, there were consumer groups that identified taste as a reason for not consuming low-alcohol beverages. The lack of taste is an important drawback of low-alcohol beverages [16] and is assumed to influence people’s perception of the quality of a given beverage.

In Hungary, the market for low-alcohol and non-alcoholic wines is a small but potentially growing target sector, including consumers who do not wish to consume large amounts of alcohol or alcohol in general because of driving, pregnancy, or a health-conscious lifestyle. To meet the needs of this sector, technologies and scientifically based practical experience are needed to demonstrate the impact of (partial) alcohol reduction on wine chemical parameters.

### 1.2. Possible Methods for Alcohol Reduction

The potential for alcohol reduction has attracted considerable interest in recent years, resulting in innovative solutions in both viticulture and oenology. In the resolution OIV-OENO 394A-2012 [17], the International Organization of Vine and Wine (OIV) listed partial vacuum evaporation, membrane techniques, and distillation as possible methods to achieve the objective of reducing part or almost all the ethanol content of wines. In addition to these, there are further methods. For example, Biyela et al. [18] and, more recently, Röcker et al. [19] showed that a glucose-oxidase enzyme derived from *Aspergillus niger* Tiegh. could be used to reduce the must sugar content through oxidation before fermentation. Röcker et al. [19] achieved a 2% *v*/*v* reduction in the alcohol content, but this resulted in increased acidity and reduced fruitiness in the wines. Some non-Saccharomyces yeast strains can ferment less sugar or divert carbon metabolism to other pathways, preventing excessive ethanol production during fermentation [20]. Furthermore, these non-conventional yeast strains can directly influence flavor formation by their own metabolic activities (production of alcohols and esters) or by releasing extracellular enzymes that convert metabolites from S. cerevisiae. Non-Saccharomyces yeast strains have also been shown to produce aromatic compounds that are not related to the activity/fermentation of many strains of S. cerevisiae, such as various monoterpenes and other terpenoid compounds [21]. However, most non-Saccharomyces yeasts cannot consume that much of the sugars; therefore, they need to be inoculated with S. cerevisiae. Reverse osmosis is a membrane-based separation technique that uses a hydrophilic semipermeable membrane, such as a hollow fiber, plate and frame, or tubular module, to create a concentration or pressure gradient, called osmotic pressure, between two solutions [22,23,24]. There are controversial findings concerning the quality of the wines made with this technology [25,26]. Osmotic distillation is a membrane-based technology in which two aqueous phases, wine (containing the volatile compounds) and water, circulate counter currently on opposite sides of a hydrophobic, hollow-fiber membrane module. The driving force of the process is the partial pressure or vapor pressure of the volatile solute in the wine and water phases [27].

Although the above-mentioned technologies have many advantages, new, innovative solutions need to be tested and specify how the wine of each grapevine cultivar could react to the treatments. For this reason, the aim of this study was to apply MASTERMIND^®^ REMOVE to reduce the alcohol content and investigate the wine analytical parameters of Pinot gris. Further aims were to explore the effects of the treatment on basic chemical components, higher alcohol, and volatile compounds, and how the concentrations of these components link to each other during the process.

## 2. Materials and Methods

### 2.1. Wine, Alcohol Reduction, and Sampling

**Figure 1:**
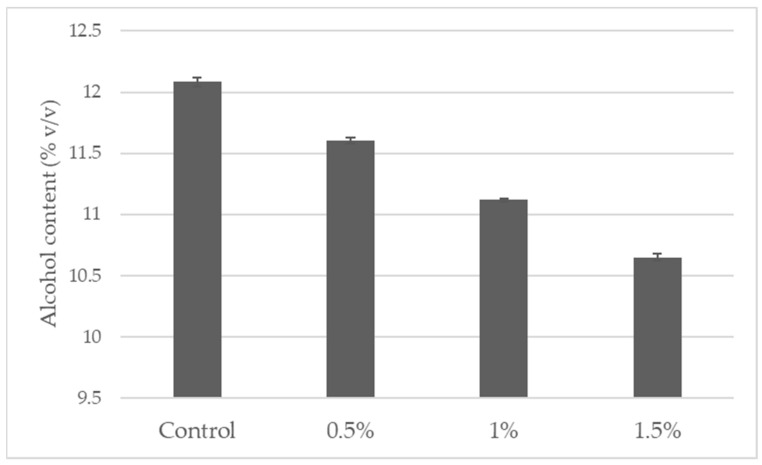
Alcohol content (% *v*/*v*) of the Pinot gris wine samples investigated in this study, where “Control” refers to 12.08% *v*/*v*, and samples with 0.5%, 1%, and 1.5% refer to an 11.6% *v*/*v*, 11.11% *v*/*v,* and 10.64% *v*/*v* alcohol content, respectively.

Pinot gris wine samples were provided by the Törley Sparkling Wine Cellar Ltd. The alcohol content of the wine was reduced from 12.08% *v*/*v* to 10.64% *v*/*v*. (Figure 1) with the MASTERMIND^®^ REMOVE membrane contactor apparatus provided by JU.CLA.S. Ltd. (Verona, Italy). The membranes were made of polypropylene hollow fibers (external diameter 1 mm, thickness 200 µm) and had a surface of 16.2 m^2^. The stripping solution was recirculating water, microfiltered (1 µm) and containing 5 g/hL of potassium metabisulphite to preserve the sulfur dioxide contained in the wine under treatment. The water was previously degassed by bubbling nitrogen through a porous plug. To avoid the dissolution of oxygen during the process, the tubes of the machine were saturated with nitrogen before the beginning of the process, which was performed at the winery temperature (around 10 °C) in a volume of 250 L for each wine; the duration of the treatment was approximately 6 h. In the operative conditions of the experiment, the volume of water employed was about 5 times the volume of the treated wines, and the stripping water at the end of the process (wines at 5% *v*/*v* alcohol) had an alcoholic degree of 2.5–3% *v*/*v*. The flow rates of wine and water were, respectively, 1.6 and 0.8 L/min, and the inlet pressure (wine) and output pressure (stripping solution) were, respectively, 1.45 and 0.95 bar [28].

During the treatment, 3 samples were obtained at the levels of 0.5%, 1%, and 1.5% alcohol reduction. At each of the 3 sampling times, two subsamples were collected from the same wine batch (i.e., biological replicates), and each subsample was analyzed three times (i.e., technical replicates). This sampling design allows for assessing both biological variability and analytical precision throughout the treatment process.

### 2.2. Wine Analytics

Wine analytics were carried out according to the following protocols. The alcohol content was determined based on the OIV-MA-AS312-01B, with a Gibertini DEE distillation and Gibertini Densimat & Alcomat hydrostatic still. The extract, which is the sum of the non-volatile, non-evaporable substances in wine (e.g., sugars, non-volatile organic acids, polyphenols, and nitrogenous substances) was determined according to the distillation of 100 mL of wine with an alcohol still. The distillate was then made up to 100 mL with distilled water. After this step, we applied a hydrostatic balance to measure the density of 100 mL of wine and then the density of the distillate added to 100 mL. Values were calculated according to Sm = Sb − Sp + 1, where Sb is the density of the wine, Sp is the distillate added to 100 mL, and Sm is the value obtained from the reference table in the study by Erdőss [29].

The reducing sugar content was determined by Schoorl titration (OIV-MA-AS311-01A), while titratable acidity was determined with acid–base titration in tartaric acid equivalents expressed as equivalent acidity (OIV-MA-AS313-01). For the pH evaluation, a combined glass electrode was applied. SO_2_ determination was carried out by titration. Determination of volatile acidity was performed by acid-based titration after steam distillation (OIV-MA-AS313-02) using a Gibertini DEE distillation apparatus. The total polyphenol content was determined spectrophotometrically with Folin–Ciocalteu reagent at 765 nm. The glycerol content determination was performed by an enzymatic method (OIV-MA-AS312-05) with a Glycerol Assay Kit (Megazyme Inc., Chicago, IL, USA), Dynamica. Succinic acid and L-boronic acid contents were determined by enzymatic methods with an L-Boronic Acid Assay Kit (Megazyme Inc., Chicago, IL, USA) and Dynamica Halo RB-10 Spectrophotometer UV/VIS, 340 nm. Proline and YAN concentrations were investigated based on the absorbance at 157 nm and 570 nm, respectively. Determinations of higher alcohol, ethyl acetate, and acetaldehyde contents were performed based on gas chromatography (column, Restek Rtx-1301 60 m × 0.25 mm; liquid injection, 10 [equilibrium]; injector, 150 °C; detector, FID 200 °C; stop time, 28.17 min.; make up, N_2_ 24 mL/min., H_2_ 32 mL/min., and air 200 mL/min.)

All the reagent were purchased from Merck KGaA (Darmstadt, Germany).

### 2.3. Statistical Evaluation

To find those components that have the most important effect, we reduced the dimensionality by performing Principal Component Analysis (PCA). Cluster analysis was run to find links between the samples and analytical parameters. Ward’s method was selected, as it minimizes the within-cluster error (variance), resulting in more compact and well-separated clusters compared to other linkage methods. Since PCA suggested separation between the groups, ANOVA was run to explore in more detail the effect of the alcohol reduction on the wine analytical parameters. Tukey’s and Dunn’s post hoc tests were run later; Levene’s test showed significance, such as n-propanol, i-butanol, i-Amylalcohol, 2-Methyl-1-butanol, Acetaldehyde, and Ethyl acetate. During treatment, the wine chemical parameters were changing simultaneously with significant correlations. To evaluate the effect of alcohol reduction on the overall wine chemical profile, multiple linear regression was carried out. Regression helps us to understand the direction and strength of the effect. Assumptions were evaluated before the run of the regression model. Residuals were tested for normality according to the Shapiro–Wilk test applied for each of the investigated wine chemical components. One variable showed a moderate deviation (normality correlation was 0.8698), while all others were higher than 0.90. Linearity and homoscedasticity were checked by plotting residuals against predicted values. Z-transformation (standardization) was applied to ensure that each variable contributed equally to the statistical analyses. This step is essential for methods such as PCA, cluster analysis, and linear regression, where unequal variable scales could otherwise bias the results. Statistical analyses were run in PAST 4.17c [30].

## 3. Results

### 3.1. Multivariate Structure of the Wine Composition (PCA Results)

**Figure 2:**
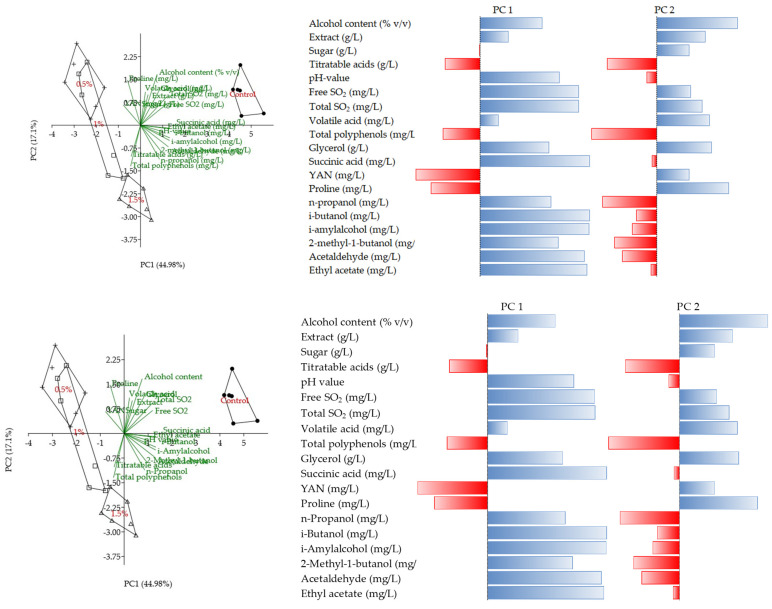
Biplot (PC1 and PC2) of the principal component analysis of chemical parameters during the partial dealcoholization of Pinot gris wines and correlations with PC1 and PC2.

According to the principal component analysis, PC1 and PC2 explained 44.98% and 17.2% of the total variance, respectively. The first five PCs explained 85.89% of the total variance. PC1 was associated with succinic acid, i-Butanol, i-Amyl alcohol, Acetaldehyde, and Ethyl acetate, while the PC2 was associated with the alcohol content and proline (Figure 2).

### 3.2. Multivariate Clustering of Treatments by Chemical Parameters

**Figure 3:**
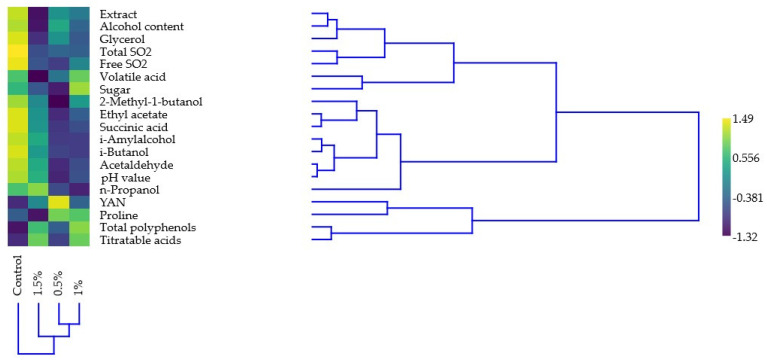
Hierarchical clustering of the treated wines and investigated chemical parameters. The color chart shows the results of Z-transformation of the original dataset.

To further explore the structure of the dataset, a hierarchical cluster analysis was performed using Ward’s method (see Figure 3). This analysis aimed to identify potential groupings among the treatments based on their multivariate similarity across all measured variables. This provides additional insights into how the samples with different alcohol contents relate to each other, going beyond the findings of the regression analysis and MANOVA. Samples with higher alcohol contents (0.5% *v*/*v* and 1% *v*/*v*) were found to be more closely related, while 1.5% *v*/*v* samples formed a distinct group. The measured variables also formed two distinct groups, where proline, YAN, total polyphenols, and titratable acids formed one distinct group, while the other group consisted of two subgroups: one consisting of the extract, alcohol content, glycerol, total and free SO_2_, volatile acids, and sugars while the other sub-group consisted of 2-Methyl-1-butanol, Ethyl acetate, Succinic acid, i-Amyl alcohol, i-Butanol, Acetaldehyde, pH, value and n-Propanol.

### 3.3. Effect of Alcohol Reduction on the Wine Chemical Composition (ANOVA)

In this study, the alcohol content of the wine was partially reduced and chemical components were monitored in three stages. According to the PCA and cluster analysis, the samples were found to be grouped; therefore, we aimed to verify this with further statistical methods. The results showed that, except for extract, sugar content, and proline content, all the other parameters showed significant differences during treatment (Table 1). Among the basic wine components, titratable acidity increased significantly from 7.78 g/L to 7.95 g/L, and a similar trend was observed for the pH value, which, although it did not show any difference between the base wine and the wine reduced by 1.5% *v*/*v*, changed significantly in the steps in between. Regarding the sulfur dioxide fractions, both the free SO_2_ and bound SO_2_ contents significantly decreased from 37.67 mg/L to 29.1 mg/L and from 113.67 mg/L to 101.67 mg/L, respectively. Alcohol reduction had a significant effect on all fermentation by-products but as with the pH value, there were some cases where there were no significant differences between the analytical parameters of the base wine and the 1.5% reduced samples. However, in the intermediate steps, there were significant differences in the 0.5% and 1% samples. Volatile acid contents showed a decrease from 0.28 mg/L to 0.23 mg/L, while in the case of the n-Propanol, i-Butanol, i-Amylalcohol, 2-Methyl-1-butanol, Acetaldehyde, and Ethyl acetate contents, there were no significant changes between the control wine and the 1.5% samples; meanwhile, the 0.5% or the 1% samples showed significant decreases. Nitrogen-related components showed similar trends. In the case of the YAN content, there was a slight increase in the 0.5% samples, and the values decreased to the same level as in the control wine, while the proline content showed no significant change during the treatment. The total polyphenol content increased from 371.67 mg/L (control wine) to 378.17 mg/L in the 1.5% wine. Significant decreases were detected in the glycerol and succinic acid contents, falling from 6.2 g/L to 5.73 g/L and from 1.79 mg/L to 1.55 mg/L, respectively.

### 3.4. Regression Models Reveal Key Trends Across Treatments

The multivariate linear regression model explained 18.84% of the total variance in the 18 variables (R^2^ = 0.188), indicating modest but meaningful relationships between the alcohol content and the investigated wine parameters. The mean square error (MSE) was 0.8485, reflecting the low average square deviation between the observed and predicted values. While this value indicates a certain degree of error in the prediction, it also provides a quantitative measure of how well the model fits the data. The MANOVA results further supported this trend, with a Wilks’ lambda of 0.0343 and a significant effect (F₍_18,5_₎ = 7.8, *p* < 0.05), and a potential multivariate relationship that requires further investigation.

### 3.5. Relationships Between the Alcohol Content and Chemical Components

In addition to the overall multivariate analysis, running individual linear regressions on the original dataset revealed a significant relation that is variable-specific (Table 2). According to the multivariate regression, the following variables had significant relationships with the alcohol content: extract, titratable acidity, free and bound SO_2_ contents, i-butanol content, total polyphenol content, glycerin content, and succinic acid content. For example, the extract had a significant and positive association (r = 0.41, *p* < 0.05) with the wine alcohol content, indicating that reduced alcohol content results in a lower extract. In contrast to this, titratable acidity showed a significant but negative correlation (r = −0.58, *p* < 0.01), with acidity increasing with a lower alcohol content from 7.78 g/l to 7.95 g/l. Both free and bound SO_2_ contents showed significant and positive trends (r = 0.65; *p* < 0.01 and r = 0.79, *p* < 0.01, respectively), suggesting noticeable reductions. The results showed that the treatment had a significant negative correlation with the total polyphenol content (r = −0.57, *p* < 0.01), while the trend was the opposite in the case of the glycerol and succinic acid contents, where the correlations were significant and positive (r = 0.75, *p* < 0.01; r = 0.42, *p* < 0.05). Of the fermentation by-products, only i-Butanol had a significant and positive correlation with the wine alcohol content (r = 0.41, *p* < 0.05).

## 4. Discussion

### 4.1. Climate Change’s Consequenses on Wine Profiles

Global climate change has a noticeable impact on the vegetative and reproductive development of plants. The recent rise in temperature is already affecting crop yields and berry composition in many wine-growing regions [4]. Climate change is one of the greatest challenges facing wine production. If the environment is too warm, the development of the grapes’ flavor and color compounds may become decoupled from sugar accumulation [9], potentially leading to wines with higher alcohol contents. The sugar composition of berries plays a key role in the quality of wines, as it determines their alcohol content. The sugar concentration of grapes varies during ripening and can be influenced by several factors, such as the environment and viticultural practices [31]. The direct winemaking consequence of an increased sugar content is an increase in the ethanol concentration of the final product, which may also lead to a decrease in the social acceptability of wines [32].

Caused by these climate change consequences and changing consumer preferences, viticulture and oenology are forced to find and adapt innovative technologies that could reduce the sugar and alcohol contents in the fruits and wine, respectively. Valera et al. [33] grouped the strategies along across the value change into four categories: (i) viticultural practices, (ii) pre-fermentation and winemaking practices, (iii) microbiological practices, and (iv) post-fermentation practices and processing technologies. Among the methods in the post-fermentation stage, Sam et al. [34] divides two groups: separation by membrane that includes reverse osmosis, pervaporation, and osmotic distillation; and separation by non-membrane techniques (thermal distillation) through vacuum distillation or a spinning cone column. For example, several studies investigated the effect of the partial or total dealcoholization according to polypropylene hollow-fiber membrane contactor [35,36,37] under vacuum [38]. In our study, similar to Motta et al. [28], MASTERMIND^®^ REMOVE with a membrane contactor was applied to reduce the alcohol content from 12.08% *v*/*v* to 10.65% *v*/*v*.

### 4.2. Wine Chemical Analysis

Several alcohol reduction methods are known; for example, it is possible to reduce the alcohol content before fermentation, during fermentation, and after fermentation. Post-fermentation reduction processes such as membrane filtration can also change the alcohol content of wines. Meanwhile, techniques during fermentation, such as the use of non-Saccharomyces yeasts, can produce more undesirable fermentation by-products such as volatile acid. This is why we consider it important to investigate the technology that can reduce the alcohol content by 1–2 *v*/*v*% without negatively affecting the sensory quality of our wine. Gambuti et al. [35] involved Merlot, Aglianico, and Piedirosso wines with alcohol contents from 13.67 to 15.46% *v*/*v* and reduced them by 2, 3, and 5% *v*/*v*. In their study, they did not find significant effects of the treatments on the sugar content (g/L), total acidity (g/L tartaric acid), dry extract (g/L), and pH. Similar results were reported by Liguori et al. [36], who applied an osmotic distillation technique and investigated Aglianico wines in five cycles of treatment from a 13% to 0.19% alcohol content. In their study, the pH value and total acidity (g/L) showed no significant differences. Lisanti et al. [37] involved two red wines of ’Aglianico’ with different initial alcohol contents (15.37 and 13.28% *v*/*v*) and partially dealcoholized at three levels (−2, −3, and −5% *v*/*v*). They also found that the sugar content (g/L), dry extract (g/L) pH value, and total acidity (g/L tartaric acid) did not change in any of the wines. In contrast with the above findings concerning the base chemical parameters, Motta et al. [28] showed that the total extract (mg/L), pH value, and titratable acidity (mg/L) were significantly changed because of the dealcoholization of a rosé, a Pelaverga, and a Barbera red wine. Our results are in accordance with both findings, as in this study, the extract and sugar content did not change. Although pH values and titratable acidy showed statistically significant differences, we consider that these changes are marginal from the from the wine sensory attribute point of view. The basic analytical parameters of the wines were determined to make sure that the alcohol reduction process does not cause any changes in the wines. The results show that the changes at this level of alcohol reduction are not remarkable.

The change in the SO_2_ fractions during the alcohol reduction showed uniform results in the literature. All former findings of Gambuti et al. [35], Lisanti et al. [37] and Motta et al. [28] indicated significant decreases. Although Lisanti et al. [36] found that the total SO_2_ content (mg/L) was decreased in the treated wines, the free SO_2_ content (mg/L) decreased only when the alcohol content was reduced from 15.37% *v*/*v*. In our study, we found that the free SO_2_ and total SO_2_ contents significantly decreased by −23.01% and −10.56%, respectively. According to our measurements, the evolution of the free SO_2_ content is influenced by the alcohol reduction process, and this is also true for the total SO_2_ content. The alcohol reduction device or treatment causes oxidation, i.e., the reduction in the SO_2_ content is the effect of oxidation due to the reduction in the alcohol content. Based on our results, it can be concluded that additional sulfuration may be necessary during the alcohol reduction process.

Concerning alcohol and more particularly i-Butanol, Lisanti et al. [37] found a decrease because of the alcohol reduction, while Liguori et al. [36] observed an alteration in the acetaldehyde concentration. In our study, no significant difference was found for the volatile acid content, and so this physical method can be used to reduce the alcohol content in wine safely and without risk. The difference in the propanol content is due to sampling error, which is within the permitted margin of error. For i-Butanol, there is a reduction, but not to an extent that would affect the formation of fruit esters. For i-amyl alcohol, the difference is also due to sampling error. The Ethyl acetate content is halved by the treatment, thus reducing the fruit character of the wines. Although the glycerol content shows a statistically significant difference (0.5 g/l reduction), it does not have a negative organoleptic effect on the wines.

The nitrogen-related compounds were investigated to determine whether there was a detectable difference between the untreated and treated wines, as these components will determine the need for fining in the future. Based on our measurements, it can be concluded that this range of alcohol reductions will not significantly reduce the wine’s need for fining. In their study, Gambuti et al. [35] did not find significant effects of the treatments on total phenolic contents (Folin–Ciocalteu index). In our study, we observed that for all polyphenols, there is no significant difference, and the reduction and oxidation ratios are neither negatively nor positively affected by the treatment.

## 5. Conclusions

Climate change is causing significant difficulties in the wine sector, for which innovative solutions can provide answers. There is a strong need for those technologies that provide the possibility of the alcohol reduction while at the same time keeping the quality of the final product. In this study, the MASTERMIND^®^ REMOVE apparatus for dealcoholization with a membrane contactor were applied to partially reduce the alcohol content of Pinot gris wines. We found that even though some of the wine chemical components changed significantly, we considered that these changes would not affect the wine sensory profile. For example, despite the decrease in the SO_2_ content, the value is still in the optimum range. Due to the reduction in the SO_2_ content due to oxidation, the impact of a possible reduction of 5–6% alcohol by volume on the parameters needs to be further investigated. Therefore, sensory analysis studies are also needed, as our experiments show that significant changes occur because of the treatment. In addition, rosé and red wine should be included in the experiment. Based on our investigation, we have concluded that partial alcohol reduction can affect the quality of the wine in terms of sensory perception and therefore we need to further investigate this process by extending it to other varieties. Further studies should be carried out in the future to obtain a comprehensive picture of the changes in aroma compounds, but the measurement of the caloric content could be a priority research direction, and, in particular, the physiological effects of compounds should be investigated.

## Figures and Tables

**Table 1:** Base chemical and fine analytical parameters of the studied Pinot gris wines.

	Control Wine	0.5%	1%	1.5%
Wine basic analysis				
Alcohol content (% *v*/*v*)	12.08 ± 0.08 _a_	11.61 ± 0.04 _b_	11.12 ± 0.03 _c_	10.65 ± 0.06 _d_
Extract (g/L)	24.1 ± 0.44	23.9 ± 0.25	23.8 ± 0.16	23.7 ± 0.55
Sugar (g/L)	1.9 ± 0.12	1.8 ± 0.05	1.9 ± 0.15	1.8 ± 0.1
Titratable acids (g/L)	7.8 ± 0.12 _b_	7.8 ± 0.11 _ab_	7.9 ± 0.08 _a_	7.9 ± 0.08 _a_
pH value	3.5 ± 0.01 _a_	3.51 ± 0.01 _bc_	3.51 ± 0.01 _b_	3.53 ± 0.01 _ab_
Sulfur dioxide fractions				
Free SO_2_ (mg/L)	37. ± 0.82 _a_	28 ± 1.26 _c_	31 ± 1.03 _b_	29 ± 1.1 _c_
Total SO_2_ (mg/L)	113 ± 1.51 _a_	103 ± 1.1 _b_	102 ± 1.03 _b_	101 ± 1.51 _b_
Fermentation by-products				
Volatile acids (mg/L)	0.28 ± 0.03 _a_	0.26 ± 0.03 _ab_	0.28 ± 0.03 _a_	0.23 ± 0.02 _b_
n-Propanol (mg/L)	38.2 ± 1.03 _a_	35.6 ± 0.67 _ab_	34.9 ± 0.67 _b_	38.7 ± 2.49 _a_
i-Butanol (mg/L)	15.8 ± 0.21 _a_	13.8 ± 0.45 _b_	13.7 ± 0.51 _b_	14.7 ± 0.43 _ab_
i-Amylalcohol (mg/L)	121.0 ± 0.43 _a_	105.6 ± 2.2 _b_	105.7 ± 1.56 _b_	114.7 ± 2.51 _ab_
2-Methyl-1-butanol (mg/L)	20.2 ± 0.15 _a_	17.0 ± 0.29 _b_	19.0 ± 2.49 _ab_	18.7 ± 0.4 _ab_
Acetaldehyde (mg/L)	36.8 ± 1.56 _a_	31.6 ± 1.22 _b_	32.4 ± 0.87 _b_	34.9 ± 0.42 _ab_
Ethyl acetate (mg/L)	49.1 ± 1.98 _a_	21.6 ± 4.52 _c_	27.6 ± 0.53 _bc_	34.6 ± 3.9 _ab_
Glycerol (g/L)	6.2 ± 0.14 _a_	5.9 ± 0.14 _ab_	5.8 ± 0.12 _b_	5.7 ± 0.24 _b_
Succinic acid (mg/L)	1.7 ± 0.05 _a_	1.3 ± 0.06 _c_	1.3 ± 0.06 _c_	1.5 ± 0.02 _b_
Nitrogen-related compounds				
YAN (mg/L)	209 ± 6.62 _b_	221 ± 3.43 _a_	212 ± 6.55 _b_	214 ± 3.56 _ab_
Proline (mg/L)	107 ± 9.38	118 ± 11.04	117 ± 16.91	102 ± 7.07
Phenolic content				
Total polyphenols (mg/L)	371 ± 3.56 _b_	374 ± 2.48 _ab_	379 ± 5.01 _a_	378 ± 4.71 _ab_

Different letters indicate significant differences at *p* < 0.05 according to Tukey’s test, and in the case of n-Propanol, i-Butanol, i-Amylalcohol, 2-Methyl-1-butanol, Acetaldehyde, and Ethyl acetate, Dunn’s post hoc test.

**Table 2:** Descriptive statistics of the multivariate regression analysis based on the original data.

	Slope	Error	Intercept	Error	r	*p*
Basic wine components						
Extract (g/L)	0.2908	0.1379	20.575	1.5682	0.4102	*p* < 0.05
Sugar (g/L)	0.0096	0.0422	1.7579	0.48	0.0483	0.8226
Titratable acids (g/L)	−0.1315	0.0387	9.3653	0.44	−0.5869	*p* < 0.01
pH value	0.0049	0.0054	3.4636	0.0611	0.1907	0.3721
Sulfur dioxide fractions						
Free SO_2_ (mg/L)	4.6966	1.1665	−21.869	13.27	0.6513	*p* < 0.01
Total SO_2_ (mg/L)	7.391	1.2151	21.264	13.823	0.7919	*p* < 0.01
Fermentation by-products						
Volatile acid (mg/L)	0.0244	0.012	−0.0148	0.136	0.3989	0.0535
n-Propanol (mg/L)	−0.0769	0.826	37.758	9.3968	−0.0199	0.9266
i-Butanol (mg/L)	0.7103	0.3369	6.4872	3.8326	0.41	*p* < 0.05
i-Amylalcohol (mg/L)	3.9473	2.517	66.954	28.634	0.3171	0.1311
2-Methyl-1-butanol (mg/L)	0.4535	0.6408	13.605	7.2903	0.1492	0.4866
Acetaldehyde (mg/L)	1.1055	0.8748	21.4	9.952	0.2601	0.2196
Ethyl acetate (mg/L)	7.4965	3.8954	−51.931	44.315	0.3796	0.0673
Glycerol (g/L)	0.3278	0.0603	2.2006	0.686	0.7571	*p* < 0.01
Succinic acid (mg/L)	0.1435	0.0658	−0.1142	0.748	0.4218	*p* < 0.05
Nitrogen-related compounds						
YAN (mg/L)	−1.0863	2.6475	226.76	30.118	−0.0871	0.6855
Proline (mg/L)	4.6535	4.9326	58.496	56.114	0.1972	0.3557
Phenolic content						
Total polyphenols (mg/L)	−5.1619	1.5732	434.53	17.897	−0.5732	*p* < 0.01

## Data Availability

The original contributions presented in the study are included in the article, further inquiries can be directed to the corresponding author.
